# Developing functional workspace for the movement of trunk circumduction in healthy young subjects: a reliability study

**DOI:** 10.1186/1475-925X-12-4

**Published:** 2013-01-11

**Authors:** Su-Chun Cheng, Chieh-Hsiang Hsu, Yi-Ting Ting, Li-Chieh Kuo, Ruey-Mo Lin, Fong-Chin Su

**Affiliations:** 1 Institute of Biomedical Engineering, National Cheng Kung University, No.1, Daxue Rd., East Dist., Tainan City, 701, Taiwan; 2 Department of Physical Therapy, Fooyin University, No.151, Jinxue Rd., Daliao Dist., Kaohsiung City, 831, Taiwan; 3 Department of Occupational Therapy, National Cheng Kung University, No.1, Daxue Rd., East Dist., Tainan City, 701, Taiwan; 4 Department of Orthopedics, National Cheng Kung University, No.1, Daxue Rd., East Dist., Tainan City, 701, Taiwan; 5 Medical Device Innovation Center, National Cheng Kung University, No.1, Daxue Rd., East Dist., Tainan City, 701, Taiwan

**Keywords:** Trunk circumduction, Motion analysis, Functional workspace

## Abstract

**Background:**

The lumbar range of motion has traditionally been used to assess disability in patients with low back disorders. Controversy exists about how movement ranges in static positions or in a single straight plane is related to the functional status of the patients. The trunk circumduction, as the result of neuromuscular coordination, is the integrated movements from three dimensions. The functional workspace stands for the volume of movement configuration from the trunk circumduction and represents all possible positions in three dimensions. By using single quantitative value, the functional workspace substitutes the complicated joint linear or angular motions. The aim of this study is to develop the functional workspace of the trunk circumduction (FWTC) considering possible functional positions in three dimensional planes. The reliability of the trunk circumduction is examined.

**Methods:**

Test-retest reliability was performed with 18 healthy young subjects. A three-dimensional (3-D) Motion Analysis System was used to record the trunk circumduction. The FWTC was defined and calculated based on the volume of the cone that was formed as the resultant scanned area of markers, multiplied by the length of the body segment. The statistical analysis of correlation was performed to describe the relation of maximal displacements of trunk circumduction and straight planes: sagittal and coronal.

**Results:**

The results of this study indicate that the movement of trunk circumduction measured by motion analysis instruments is a reliable tool. The ICC value is 0.90-0.96, and the means and standard deviations of the normalized workspace are: C7 0.425 (0.1162); L1 0.843 (0.2965); and knee 0.014 (0.0106). Little correlations between the maximal displacement of trunk circumduction and that of straight planes are shown and therefore suggest different movement patterns exist.

**Conclusions:**

This study demonstrates high statistical reliability for the FWTC, which is important for the potential development as the functional assessment technique. The FWTC provides a single integrated value to represent angular and linear measurements of different joints and planes. Future study is expected to carry out the FWTC to evaluate the amount of workspace for the functional status of patients with low back injuries or patients with spinal surgery.

## Background

The assessment of low back disorder (LBD) remains a challenge for clinicians. Numerous studies have described the controversies of making diagnoses for patients with LBD [1]. Diagnosis by examination of patho-anatomical structures has been found to inconsistently relate to the clinical symptoms, and a high false rate has been reported [1-7]. Measuring the functional status of the subjects with LBD in addition to other forms of medical assessment has been reported as having great advantages in quantifying the impact of LBD on daily activities, and can serve to guide clinicians for treatment purposes [8-11].

The lumbar range of motion has traditionally been used to assess disability in patients with LBD. Therapists commonly measure the range of the cardinal planes in static positions. These measures have been inconclusive in determining the pathoanatomic structures or the functional limitations [12,13]. The impaired structures automatically move the spine to avoid pain in order to complete the required tasks. The end position may not be limited, but the dynamic movement pattern would alter. An increasing number of studies have been carried out to evaluate lumbar functions by dynamic motion assessment. Several studies have investigated the lumbar movements on a single plane related to the presence of low back pain, but the results remain inconsistent [14-16]. Furthermore, the single-plane range of lumbar motion shows little relationship to self-reported scores and work status in patients with subacute and chronic LBD [17-19]. In the Parnianpour study [20], the authors addressed the importance of spinal movement graphics to explain spinal dysfunctions. They stated that performing spinal movements in functional activities was a continuous task that needed anatomical structures to distort and recoil in different directions repeatedly.

The lumbar spine is a multi-articular region supporting movements on three planes to perform daily activities [21]. Due to natural curvature or facet orientations of the spine, the spinal coupling motion is defined that the lumbar spine move in a primary plane in accompany with movements in a secondary plane. Studies in vivo have described the different patterns of coupling motion between normal and injured lumbar spines [22-24].

Motion analysis instrumentation can track three-dimensional (3-D) dynamic spinal movement and measure the spatial and temporal characteristics of that movement. The circumduction is a result of neuromuscular coordination and represents possible extreme positions in three dimensions. There are two advantages for using the trunk circumduction as a functional task, one for dynamic motions and the other for motion coupling of the spine. A three dimensional movement has been described in a study investigating the movements of the thumb and in a study investigating the movement of hemiparesis patients. The determination of the workspace is therefore established to represent the volume of movement configuration [25-27]. The concept of the workspace has been described in parallel kinematic studies as the distance a robot can reach [28,29], or the bimanual seated positions of patients with spinal cord injuries [30]. Su et al [25] investigated the workspace by circumduction of the thumb and concluded that the 3-D workspace could potentially be used to evaluate the motion of joints affected by disease or injury.

Based on the concept of the three-dimensional (3-D) space within which the trunk can circumduct, hereafter the workspace of the trunk, the current study proposes a quantitative method for evaluating the degree of lumbar active movement in three dimensions, and the test–retest reliability of this method. This study incorporates the following key features that add to its clinical implications: (1) the trunk motion is described in a three dimensional space and is referenced to an anatomically relevant local coordinate system; (2) statistical analysis is applied to determine the reliability of the measures used to describe motion; and (3) correlations of the circumduction among three orthogonal axes are performed with the movement of a single plane, for example sagittal and coronal movements, respectively. In this way, the associations between the composite movement and single planes can be established.

## Methods

### Subjects

Ethical approval was obtained from National Cheng Kung University Hospital (NCKUH). All subjects gave informed consent. Eighteen young and healthy subjects (8 males and 10 females; aged 21-28 years) recruited by local advertising participated in this study. The inclusion criteria were that the subjects should not have suffered any back pain or back related leg pain, injury, or surgery. Subjects participated in a reliability test and were evaluated on two different test days, with seven-day intervals between test days. On each test day, the subjects performed trials of the trunk circumduction tests. The data from the 18 subjects were combined to determine an ideal trunk circumduction.

### Instrumentation

A three-dimensional (3-D) Motion Analysis System with a set of 8 opto-electric cameras supported by EVaRT^TM^ 4.2 software (Motion Analysis Corporation, Santa Rosa, CA, USA) was used to record the trunk circumduction at a sampling rate of 60Hz. The instrument was capable of tracking the movement of markers in three dimensions within a 0.1% error of the field within the field of view. This system incorporated eight video cameras, video processors, a Sun workstation and a personal computer.

### Experimental procedures

**Figure 1:**
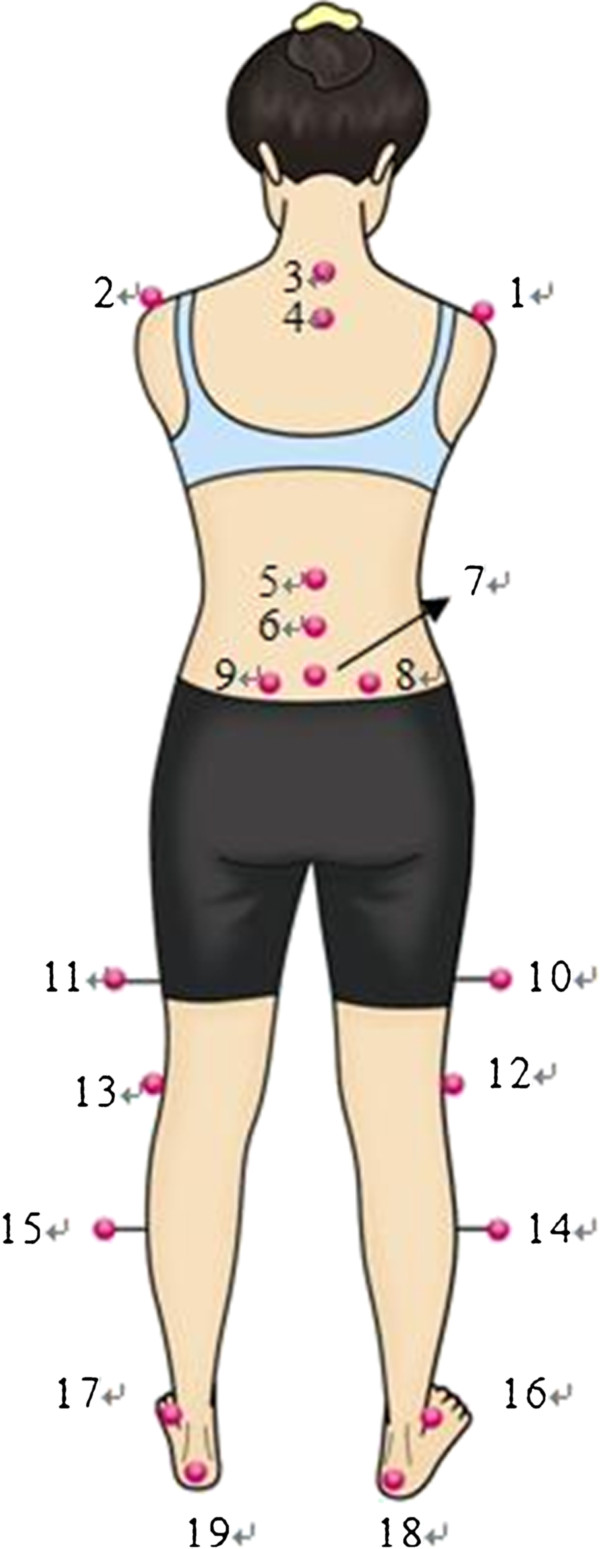
**The marker settings of the trunk and lower extremities. **Marker 1and 2: right and left acrominon; marker 3: C7; marker 4: T4; marker 5: L1; marker 6: L3; marker 7: L5; marker 8 and 9: right and left posterior superior iliac spines; marker 10 and 11: right and left middle thigh; marker 12 and 13: right and left lateral knees; marker 14 and 15: right and left middle lower legs; marker 16 and 17: right and left of the base of the second metatarsal bones; marker 18 and 19: right and left calcaneus.

After obtaining the participants’ consent and providing a brief explanation of the study, the anthropometric parameters were assessed. A set of twenty-one retro-reflexive markers, 10 mm in diameter, were attached with adhesive tapes to the lower limbs, pelvis and spinal landmarks by a research assistant (Figure 1). The landmarks of the trunk comprised the C7, T4, L1, L3, L5 spinous processes, and bilaterally, the acromions, and the anterior superior iliac spines (ASISs) and posterior superior iliac spines (PSISs). The landmarks of the lower limbs comprised bilaterally 1/2 thighs, lateral femoral epicondyles, 1/2 lower legs, lateral malleoli, and heels. The landmarks of two ASISs and PSISs served to define a local coordinate system which differentiated movement within the spine compared to total body movement.

**Figure 2:**
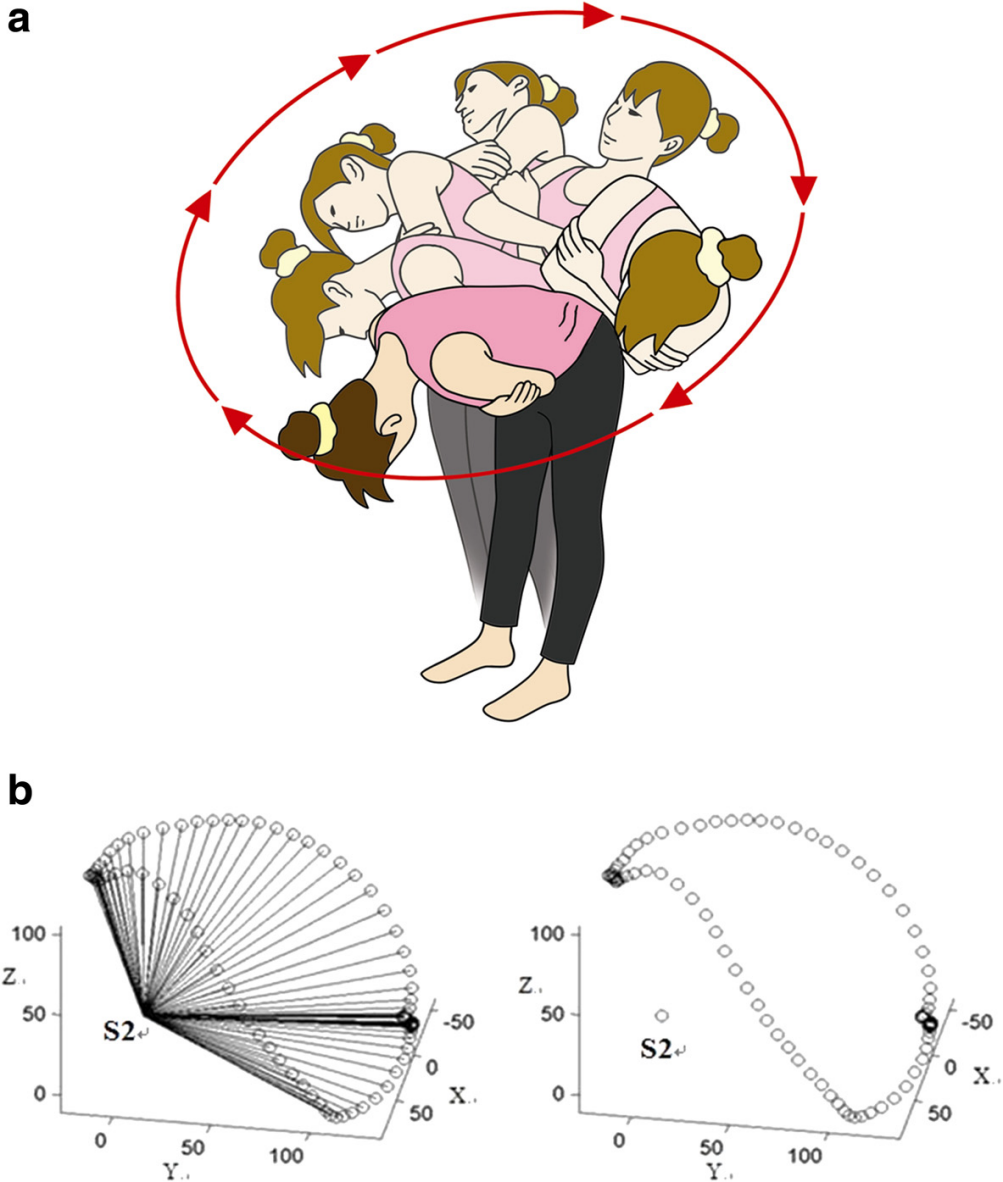
**a. Diagram of movements of trunk circumduction. b**. An example of illustration of the L1 functional workspace of trunk circumduction.

Following the anthropometric measurements, each participant went through a detailed practice of motions. Laboratory testing consisted of a series of movements randomly decided by tossing a coin, including sagittal flexion and extension, coronal sidebendings, and the trunk circumductions. Particularly, the movement of trunk circumduction was introduced and followed by a brief practice session. Movement trials were performed and recorded separately from the video-computer systems. At first, the participants stood with feet shoulder-width apart, knees straight and arms held across the chest at all times. For a sagittal movement, the participants bent forward as far as possible, paused for 3 seconds and then rose to an upright position, paused for 3 seconds, continued to bend backwards as far as possible, paused for 3 seconds, and then came back to the upright position. For a coronal movement, the participants bent sideways to one side as far as possible with a pause of 3 seconds before rising to the upright position, pausing for another 3 seconds and continuing to bend to the other side, with another pause before coming back to the upright position. For a circumduction, the participants bent the trunk to the self-determined maximal flexion. Then the participant moved the upper body to follow a circular pathway, that is, from flexion to sidebending and then extension to the other side for sidebending and flexion back to the beginning point (Figure 2). While performing the trunk circumduction movements, the subjects tucked in their chin to avoid dizziness or stress on the neck. Subjects were asked to keep the lower limbs as straight as possible. Subjects performed two sides of trunk circumductions, three trials for each side. The second trial was used for analysis, with considering that subjects may not feel confident in the first trial and may move too fast to perform extreme positions in the third trial.

For the post-processing procedure, a MATLAB® program was developed to compute the workspace of the circumduction. The workspace was defined as the volume of the cone that was formed as the resultant scanned area of markers by the circumduction, multiplied by the length of the body segment. The scanned area was defined as the area intergration of the tracking pathway of the selective markers from the beginning point and back to the original. This study calculated the workspace of C7, L1 and the knee region relative to the local pelvic system. The assessment of the validity of functional workspace was provided by comparison of by manual calculation or by the MATLAB program for the tracking pathway of the mobile mini-train on the round railway. The height from the train marker to the floor marker was 402.8 mm and the diameter of the round track was 425.6 mm. The difference of volume calculation by the Matlab program compared to manual calculation was 0.97%.

### Transformations between the laboratory and the local coordinate system

A point V in the space was measured by motion capture system and expressed by the vector **v**_*G*_ with respect to the laboratory coordinate system, *X-Y-Z*. For a local coordinate system, *x-y-z*, with the origin at **r** offset from the laboratory coordinate system, the rotation matrix to map from the local coordinate system to the laboratory coordinate is expressed by **R**. For a point V in the space, the mapping between the laboratory coordinate system and the local coordinate system is expressed by

$$v_{G}=Rv_{L}+r$$

where **v**_*L*_ is the vector of point V expressed in the local coordinate system. Then, the description **v**_**L**_ with respect to the local coordinate system is described by

$$v_{L}=R^{T}\left(v_{G}-r\right)$$

Through transformation between the laboratory coordinate system and the local coordinate system, the trunk and the lower extremity trajectory were expressed related to the pelvis in which later data were referenced for further investigation.

### Statistical analysis

Statistical analyses were undertaken with SPSS^TM^ (Version 17.0). Mean and standard deviations of FWTC and and maximal displacements were calculated. The test-retest reliability of functional workspace was carried to determine whether the vider-based motion system was a reliable tool. Pearson correlation was used for correlation analysis among the maximal displacements of the trunk movements.

## Results

The maximal volume of trunk circumduction for trials on the first test day and repeated measurement on the second test day are shown in Table 1. The mean and standard deviations of the workspaces for C7, L1 and knees, which were normalized by segments, are presented. The ICC values of the repeated measurements between the two different test days are in the range of .89 to .96. The results indicate that the quantitative tool as measured by the video-based motion analysis system is a reliable tool for assessing the circumduction range of motion of the trunk.

**Table 1:** The repeated measurement of the maximal volume workspace of the trunk circumduction in 18 healthy young subjects

**Subject**	** C7**			** L1**			** knee**		
	**first day**	**second day**	**Mean (SD)**	**first day**	**second day**	**Mean (SD)**	**first day**	**second day**	**Mean (SD)**
1	0.515	0.570	0.542 (0.0388)	0.418	0.468	0.443 (0.0350)	0.004	0.002	0.003 (0.0010)
2	0.423	0.368	0.396 (0.0391)	0.853	0.915	0.884 (0.0437)	0.014	0.018	0.016 (0.0028)
3	0.622	0.562	0.592 (0.0429)	1.323	1.388	1.356 (0.0460)	0.012	0.013	0.012 (0.0011)
4	0.499	0.554	0.526 (0.0386)	0.876	0.956	0.916 (0.0569)	0.010	0.012	0.011 (0.0017)
5	0.405	0.354	0.380 (0.0360)	0.618	0.564	0.591 (0.0384)	0.004	0.011	0.008 (0.0046)
6	0.658	0.596	0.627 (0.0438)	0.974	0.873	0.923 (0.0713)	0.022	0.012	0.017 (0.0076)
7	0.617	0.543	0.580 (0.0524)	0.846	0.737	0.791 (0.0768)	0.013	0.008	0.010 (0.0035)
8	0.283	0.314	0.298 (0.0220)	0.848	0.905	0.877 (0.0403)	0.024	0.019	0.022 (0.0039)
9	0.446	0.451	0.448 (0.0038)	1.148	1.127	1.137 (0.0152)	0.009	0.014	0.011 (0.0036)
10	0.377	0.405	0.391 (0.0199)	0.757	0.853	0.805 (0.0680)	0.005	0.009	0.007 (0.0029)
11	0.538	0.469	0.504 (0.0483)	1.054	1.096	1.075 (0.0297)	0.010	0.013	0.012 (0.0016)
12	0.417	0.357	0.387 (0.0423)	1.143	0.730	0.937 (0.2921)	0.045	0.056	0.050 (0.0082)
13	0.263	0.279	0.271 (0.0110)	1.278	1.378	1.328 (0.0710)	0.021	0.022	0.022 (0.0007)
14	0.393	0.395	0.394 (0.0016)	0.611	0.618	0.614 (0.0052)	0.015	0.016	0.016 (0.0004)
15	0.280	0.249	0.265 (0.0216)	0.604	0.554	0.579 (0.0354)	0.006	0.004	0.005 (0.0016)
16	0.322	0.403	0.363 (0.0570)	0.624	0.681	0.652 (0.0403)	0.016	0.011	0.014 (0.0031)
17	0.336	0.328	0.332 (0.0059)	0.961	1.032	0.997 (0.0499)	0.015	0.012	0.014 (0.0025)
18	0.409	0.265	0.337 (0.0102)	0.617	0.487	0.489 (0.0919)	0.009	0.008	0.009 (0.0001)
Mean	0.434	0.415		0.879	0.853		0.014	0.014	
SD	0.1202	0.1123		0.2569	0.2781		0.0097	0.0114	

**Figure 3:**
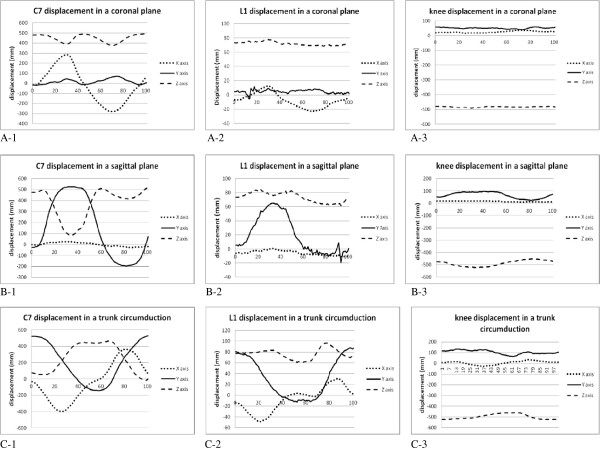
Displacements of C7, L1 and knee in three orthogonal axes of a coronal movement (A-1~A-3), a sagittal movement (B-1~B-3), and a trunk circumduction (C-1~C-3).

The maximal displacements of C7, L1 and knees performed on straight planes and trunk circumduction are presented in Table 2. In the movements on the straight planes, the primary movement occurred on the Y axis when performing a sagittal movement, and on the X axis for a coronal movement. During the trunk circumduction, the movements involved three dimensional motions on the X, Y and Z axes (Figure 3). The correlations of the displacements among the three orthogonal axes performed in a trunk circumduction with displacements of the movement of a single plane are shown in Table 3. The circumduction is least related to the sagittal movement on the Y axis, or to the coronal movement on the X axis.

**Table 2:** The maximal displacements in X, Y, Z axis of C7, L1, and knee markers in performing in straight planes and trunk circumduction (normalized by body segments C7-S2, L1-S2, and knee-S2, respectively)

	**Coronal movement**	**Sagittal movement**	**Trunk circumduction**
C7 X axis	1.12 (0.334)	0.06 (0.018)	1.79 (0.193)
L1 X axis	0.44 (0.231)	0.50 (0.389)	1.77 (0.432)
knee X axis	0.07 (0.061)	0.09 (0056)	0.23 (0.075)
C7 Y axis	0.09 (0.050)	1.50 (0.406)	1.53 (0.115)
L1 Y axis	0.28 (0.151)	1.46 (0.448)	1.73 (0.223)
Knee Y axis	0.03 (0.010)	0.20 (0.066)	0.23 (0.109)
C7 Z axis	0.26 (0.097)	1.13 (0.307)	1.35 (0.154)
L1 Z axis	0.63 (0.334)	0.85 (0.335)	1.05 (0.346)
Knee Z axis	0.02 (0.012)	0.30 (0.171)	0.17 (0.037)

**Table 3:** Correlations between maximal displacements of trunk circumduction and those of coronal and sagittal planes

**3A**		**Trunk circumduction**		
		**C7 X axis**	**L1 X axis**	**knee X axis**
	C7 X axis	−0.133	−0.315	0.132
Coronal movement	L1 X axis	−0.158	−0.382	−0.027
	knee X axis	−0.038	−0.02	0.067
	C7 X axis	−0.415	−0.395	−0.163
Sagittal movement	L1 X axis	0.17	−0.078	−0.006
	knee X axis	0.129	−0.082	0.108
**3B**				
		**C7 Y axis**	**L1 Y axis**	**knee Y axis**
	C7 Y axis	−0.108	−0.402	0.024
Coronal movement	L1 Y axis	−0.515^a^	−0.464	0.271
	knee Y axis	0.065	0.028	−0.261
	C7 Y axis	0.107	−0.147	−0.026
Sagittal movement	L1 Y axis	0.027	−0.044	−0.033
	knee Y axis	0.029	−0.192	−0.241
**3C**				
		**C7 Z axis**	**L1 Z axis**	**knee Z axis**
	C7 Z axis	0.075	−0.175	−0.116
Coronal movement	L1 Z axis	0.434	0.271	−0.264
	knee Z axis	−0.226	−0.354	0.523^a^
	C7 Z axis	−0.213	−0.34	−0.017
Sagittal movement	L1 Z axis	−0.397	−0.379	−0.365
	knee Z axis	−0.292	−0.32	−0.381

3A represents relationships of movements in X axis; 3B in Y axis; 3C in Z axis.

^a^*p<0.05.*

## Discussion

Impairments of appropriate functions of the lumbar spine result in a limited range and avoidance of painful movement after injuries. Controversy exists if motion assessment can objectively measure the loss of lumbar spine motion or the movement pattern after injuries. Past studies which measured lumbar motion usually assessed the trunk movement on a single plane or the static end-range position and poor relationship to functions were indicated [17]. Based on the concept of the three dimensional space in which the lumbar spine can move, a new method of functional workspace is first developed to aid clinical measurement of lumbar spine motion and to potentially determine three dimensions of lumbar impairment resulting from disease and injury.

Before developing the assessment of the FWTC and applying to patients with LBP, the test-retest reliability of the trunk circumduction is verified. Based on the results of this study, the ICC values for our reliability test demonstrate high reliability and indicate low variability of the data of the maximal lumbar workspace. Due to the measurement from the three dimension space, comparing data with prior studies with two dimension space may not be possible. In this study, the maximal displacements are least correlated through three orthogonal axes of circumduction and movements occurring on single planes. The results suggest the movement patterns of trunk circumduction are independent to those occurring in single planes. The circumduction moves the trunk in a combined pattern; that is, a different amount of combinations in any time frame with sidebending, rotation and with flexion or extension which is different from the movement of the straight planes.

The current study does not use restraints to control the movement of the lower extremities in performing trunk circumduction as well as other planar motions, as was done in a previous study [31]. Subjects were just told to keep the knees as straight as possible when performing the tests. The general idea was that subjects performing trunk motions would naturally make compensatory motions of the lower extremities in order to keep the center of pressure within the base of support. This study not only evaluates the effect of the trunk but also that of the lower extremities on the trunk circumduction. The tracking pathway of the surface landmarks measured from the laboratory coordinate system transforms into the local pelvic system. The origin of the pelvic coordinate system is the S2. The advantage of the data referring to the local pelvic system is similar to the conditions of viewing the landmarks from the pelvis, and would not be confounded by movements of the surrounding landmarks. Using this analysis, the movement of the lumbar and lower extremities could be assessed separately. This thought is in accordance with the study of Mannion et al [32], who measured the range of motion and disability scores after lumbar decompression surgery and highlighted the importance of measuring the lumbar and hip ranges of motion separately. Similar approaches are found in Boninger et al.’s study of the description of upper extremity motions in wheelchair propulsion [33].

There are several advantages of applying the movement of trunk circumduction to measuring the workspace. Trunk circumduction provides three dimensional through-range patterns to evaluate mechanical abnormities to aid diagnosis and treatment. The workspace uses the volume formula to calculate the maximal available range and to come to a reliable tool to describe the movement integrity. However, the use of trunk circumduction as a measurement of lumbar motion has its drawbacks. Due to the complexity of circumduction, subjects need to concentrate and to practice the movement several times before formal testing. Setting up the motion analysis system, attaching reflective markers to the subjects and analyzing the data are also time-consuming. Further studies could use other time-saving instruments to measure the workspace of the trunk circumduction, such as electromagnetic devices. Another limitation is that the subjects in this study are young and healthy and the sample size is small. Further studies could focus on larger samples of other age groups or patients with low back disorder.

## Conclusions

This study indicates that the movement cycle of trunk circumduction is more complicated than movements on straight planes. The movement patterns of trunk circumduction are independent to those occurring in single planes. The maximal lumbar workspace by trunk circumduction demonstrates high reliability of the data and can potentially evaluate the motion of joints affected by disease or injury.

## Competing interests

The authors declare that they have no competing interests.

## Authors’ contributions

SCC, CHH, YTT designed research, performed experiments, analysed data, discussed the results, and involved in drafting the manuscript. LCK, RML participated in the research designs of the study. FCS made contributions in interpretation of data and coordination. All authors read and approved the final manuscript.
